# Role of Adjuvant Chemotherapy After Surgical Resection of Paraaortic Lymph Node Metastasis from Colorectal Cancer—A Multicenter Retrospective Study

**DOI:** 10.1245/s10434-024-16537-6

**Published:** 2024-11-18

**Authors:** Hiroaki Nozawa, Sono Ito, Kazuhito Sasaki, Koji Murono, Shigenobu Emoto, Yuichiro Yokoyama, Shinichi Yamauchi, Yusuke Kinugasa, Yoichi Ajioka, Soichiro Ishihara

**Affiliations:** 1 Department of Surgical Oncology, Graduate School of Medicine, The University of Tokyo, Tokyo, Japan; 2 Gastrointestinal Surgery, Institute of Science Tokyo, Tokyo, Japan; 3 Department of Surgery, Soka Municipal Hospital, Saitama, Japan; 4 Division of Molecular and Diagnostic Pathology, Graduate School of Medical and Dental Sciences, Niigata University, Niigata, Japan; 5 Study Group for Paraaortic Lymph Node Metastases, The Japanese Society for Cancer of the Colon and Rectum, Tokyo, Japan

**Keywords:** Colorectal cancer, Paraaortic lymph node metastasis, Adjuvant chemotherapy, 5-fluorouracil, Oxaliplatin

## Abstract

**Background:**

Surgical removal of metastasized paraaortic lymph nodes (PALNs) can prolong the survival of certain patients with colorectal cancer (CRC). However, the role of postoperative chemotherapy in such patients remains unknown.

**Patients and Methods:**

This multicenter retrospective study examined 97 patients with PALN metastasis from CRC who underwent surgical resection at 36 centers in Japan between 2010 and 2015. On the basis of adjuvant chemotherapy (AC) after the lymphadenectomy, patients were classified into non-AC and AC groups (27 and 70 patients, respectively). After the exclusion of patients receiving irinotecan, the latter group was further categorized into 5-fluorouracil (5-FU) and oxaliplatin (L-OHP) subgroups (14 and 52 patients, respectively) according to the use of L-OHP. Background characteristics and postoperative survival were compared among the groups.

**Results:**

Marked differences were not seen in background characteristics, except for neoadjuvant treatment, between the non-AC and AC groups. The AC group exhibited better recurrence-free survival (RFS; *p* = 0.009) and overall survival (OS; *p* = 0.040 by the Wilcoxon test) than the non-AC group. However, RFS and OS of the 5-FU group did not differ from those of the L-OHP group (*p* = 0.73 and *p* = 0.92 by the Wilcoxon test, respectively).

**Conclusions:**

AC may be associated with improved prognosis of patients after the removal of PALN metastasis from CRC, but L-OHP did not offer additional survival benefits. Prospective studies comparing non-AC with 5-FU- and L-OHP-based AC are needed to confirm these findings.

**Supplementary Information:**

The online version contains supplementary material available at 10.1245/s10434-024-16537-6.

Colorectal cancer (CRC) is one of the common malignancies worldwide.^1^ Approximately 50% of patients with CRC are diagnosed with distant metastasis at initial presentation or metachronously.^2–4^ Paraaortic lymph node (PALN) metastasis is observed in only 2% of patients with CRC,^5,6^ and is also considered as distant metastasis in the classification by the American Joint Committee on Cancer and the Japanese Society for Cancer of the Colon and Rectum (JSCCR).^7,8^ As the paraaortic lymphatic flow drains easily into the thoracic duct, metastasis in PALNs is considered a systemic disease and is therefore not an indication for surgery.

PALN resection was first reported more than 60 years ago,^9^ and its feasibility and indication for PALN metastasis from CRC have been examined by different studies.^10–14^ The link between complete surgical resection of metastasized PALNs and favorable survival has been documented in numerous studies using both surgical and nonsurgical cases of PALN metastasis together.^15–21^ However, there is a scarcity of studies exploring the role of adjuvant chemotherapy (AC) after lymphadenectomy of metastasized PALNs from CRC owing to the rarity of such cases. No investigations have so far confirmed the significant survival impact of AC.^14,17,19,22^ Of note, two Japanese groups independently reported that the 5-year overall survival (OS) rate of patients receiving AC after surgery for PALN metastasis from CRC was 31%–36%, whereas none survived for this duration without AC.^16,17^ A recent multicenter study in Japan showed that patients with CRC who underwent surgery for PALN metastasis and did not receive postoperative chemotherapy tended to exhibit unfavorable survival outcomes [hazard ratio: 1.53 for recurrence-free survival (RFS) in R0 cases, *p* = 0.07].^23^

In this study, the survival of patients who received AC and those who did not receive the treatment was compared using patients who underwent curative-intent surgery for PALN. The patients were selected from the same study cohort mentioned above.^23^ Patient survival was further compared according to the AC regimen.

## Patients and Methods

### Patients

Patients who underwent curative surgery (R0 or R1 resection) for PALN metastasis from primary CRC between 2010 and 2015 at 36 centers in Japan were included in this retrospective study.^23^ They were diagnosed with PALN metastasis by at least one of the following: (1) enlarged PALN on preoperative computed tomography (CT) scan, (2) increased fluorodeoxyglucose uptake into PALN on positron emission tomography, and (3) intraoperative enlargement of PALN.^23^ Resected PALNs localized from the aortic hiatus to the aortic bifurcation, and were resected by dissection or pick-up which depended on the hospital.^23^ Both primary CRC and PALN metastasis were histologically proven. False-positive cases, noncurative cases in which metastatic lesions diagnosed preoperative and/or intraoperatively remained after PALN resection were not excluded. Patients with unavailable information on postoperative chemotherapy or those treated with drugs other than oral or intravenous 5-fluorouracil (5-FU), such as cetuximab alone, and those lacking survival data were further excluded.

Patients were first classified into non-AC and AC groups according to AC after PALN resection. Furthermore, on the basis of whether the AC regimen included oxaliplatin (L-OHP), the latter group was in turn categorized into 5-FU (without L-OHP) and L-OHP subgroups. Although irinotecan is not considered to confer a significant improvement in survival in the adjuvant setting in stage III CRC,^24^ the drug may exert an unreported meaningful influence on survival in CRC with PALN metastasis. Thus, patients receiving irinotecan-containing regimens were excluded from the analyses comparing 5-FU and L-OHP subgroups. The implementation, regimen, and dosage of AC after PALN resection was at the discretion of individual center.

This study was approved by the JSCCR ethical committee (95-1), the institutional review board of the University of Tokyo (2021157NIe), and that of Institute of Science Tokyo (formerly Tokyo Medical and Dental University, M2020-357).

### Evaluated Parameters and Outcome Measures

Hospitals were dichotomized around the median number of patients into the low- and high-volume centers. Age, sex, primary tumor location, histological type, serum carcinoembryonic antigen (CEA) and carbohydrate antigen (CA) 19-9 levels, pathological tumor depth (pT) and regional lymph node metastasis (pN) of the primary tumor, metastasis to other organs, timing of PALN metastasis (synchronous or metachronous), preoperative chemotherapy before PALN resection, number of metastasized PALNs, and complications after PALN resection graded according to the Clavien–Dindo classification^25^ were the baseline variables reviewed. In addition, TNM staging of CRC was defined according to the JSCCR classification.^8^

Follow-up plan after PALN resection was dependent on the individual hospital. Recurrence after curative surgery for PALN metastasis and treatment modalities for the recurrence were searched. RFS was defined as the time between the date of PALN resection and recurrence, and OS was defined as the time between PALN resection and death from any cause.

In this study, preoperative and perioperative parameters of PALN resection, RFS, and OS were compared between the non-AC and AC groups, as well as between the 5-FU and L-OHP groups.

### Statistical Analysis

An unpaired *t* test or Mann–Whitney *U* test was used to compare the continuous variables. Fisher’s exact test or Chi square test with Yates’ correction where appropriate was utilized to compare the categorical data. The Kaplan–Meier method was used to draw the RFS and OS curves, and they were compared using the log-rank test and generalized Wilcoxon test. The hazard ratio and associated 95% confidence intervals for treatment effects on RFS or OS were calculated for each subgroup and presented as forest plots. The patients were subdivided into **-**pT3 and pT4 regarding pT and into **-**pN1 and pN2- regarding pN in consideration of the number of subpopulations. Moreover, patient age and number of positive PALNs were dichotomized on the basis of their median values in forest plot analyses. All analyses were performed using the JMP 17.2.0 software program (SAS Institute, Inc., Cary, NC, USA), and a *p* value of < 0.05 was considered significant.

## Results

### Patient Selection

**Fig. 1:**
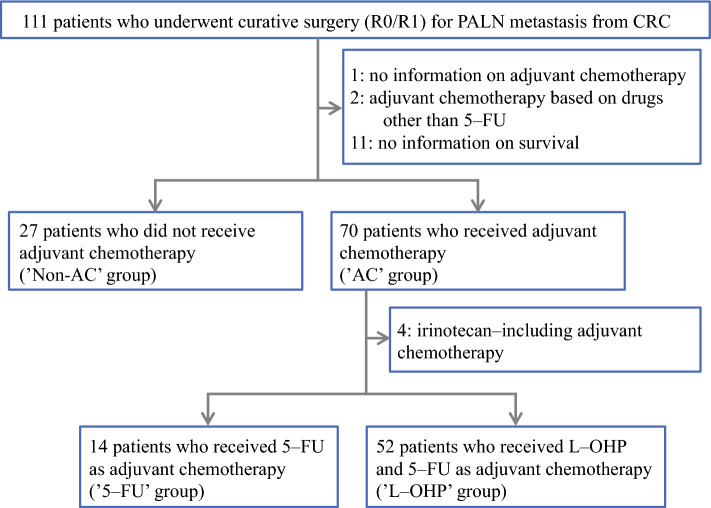
Flow diagram of the study cohort. The patients were first classified into the non-AC and AC groups according to adjuvant chemotherapy, and the latter was further categorized into the 5-FU and L-OHP subgroups according to the adjuvant chemotherapy regimen *PALN* paraaortic lymph node, *CRC* colorectal cancer, *AC* adjuvant chemotherapy, *5-FU* 5-fluorouracil, *L-OHP* oxaliplatin

Of the 111 patients who underwent curative surgical resection for PALN metastasis from CRC, 14 were excluded from the study and the remaining were classified into non-AC (27 patients) and AC groups (70 patients). In addition, 14 patients were placed in the 5-FU group and 52 patients in the L-OHP group after excluding four patients who received 5-FU and irinotecan as AC (Fig. 1). The 97 patients analyzed were registered from 25 out of 36 participating hospitals. According to the median number (3) of patients (range: 1―12) per hospital, 14 hospitals were classified into the low-volume center group (3 patients or less), and 11 were the high-volume center group (4 patients or more).

### Comparison of Baseline Characteristics and Prognosis According to Adjuvant Chemotherapy

Background characteristics of the non-AC and AC groups are presented in Table 1. More patients received neoadjuvant therapy in the non-AC group than in the AC group (44% versus 21%, *p* = 0.015). In addition, the non-AC group comprised more patients who underwent dissection for PALN removal than the AC group (81% versus 51%, *p* = 0.013). Significant differences were not perceived in other clinical variables or pathological parameters related to primary CRC and PALN metastasis. The frequency of severe complications (Clavien–Dindo classification grade 3) after PALN resection was comparable between the two groups.

**Table 1:** Baseline characteristics of patients according to adjuvant chemotherapy after paraaortic lymph node resection

Variable		Non-AC (*n* = 27)	AC (*n* = 70)	*p*
Hospital	Low-volume center	6 (22%)	18 (26%)	0.93
	High-volume center	21 (78%)	52 (74%)	
Age, years	Median (IQR)	65 (59–70)	63 (53–67)	0.12
Sex	Male	12 (44%)	42 (60%)	0.17
	Female	15 (56%)	28 (40%)	
Primary tumor location	Colon	10 (37%)	30 (43%)	0.60
	Rectum	17 (63%)	40 (57%)	
Histology	Differentiated	23 (85%)	62 (89%)	0.91
	Others	4 (15%)	8 (11%)	
pT	pT1	1 (4%)	0 (0%)	0.13
	pT2	2 (7%)	0 (0%)	
	pT3	17 (63%)	35 (50%)	
	pT4a	2 (7%)	24 (34%)	
	pT4b	5 (19%)	11 (16%)	
pN	pN0	6 (22%)	3 (4%)	0.083
	pN1	7 (26%)	12 (17%)	
	pN2	6 (22%)	22 (32%)	
	pN3	8 (30%)	33 (47%)	
Timing of PALN metastasis	Synchronous	19 (70%)	48 (69%)	0.94
	Metachronous	8 (30%)	22 (31%)	
Other metastasis at initial presentation	None	23 (85%)	61 (87%)	0.75
	Other distant lymph nodes	0 (0%)	1 (1%)	1.00
	Liver	2 (7%)	6 (9%)	1.00
	Lung	2 (7%)	1 (1%)	0.19
	Peritoneum	0 (0%)	3 (4%)	0.56
	Ovary	0 (0%)	1 (1%)	1.00
	Unknown	1 (4%)	0 (0%)	N/E
Neoadjuvant treatment for PALN metastasis	No	15 (56%)	56 (79%)	0.015
	Yes	12 (44%)	14 (21%)	
Technique of PALN resection	Dissection	22 (81%)	36 (51%)	0.013
	Pick-up	5 (19%)	34 (49%)	
Number of positive PALNs	Median (IQR)	2 (1–6)	2 (1–3)	0.39
Complications after PALN resection *	No	21 (78%)	61 (87%)	0.41
	Yes	6 (22%)	9 (13%)	

^*^Clavien-Dindo classification grade 3 or higher

*AC* adjuvant chemotherapy, *IQR* interquartile range, *Differentiated* differentiated adenocarcinoma, *PALN* paraaortic lymph node, *N/E* not evaluated

**Fig. 2:**
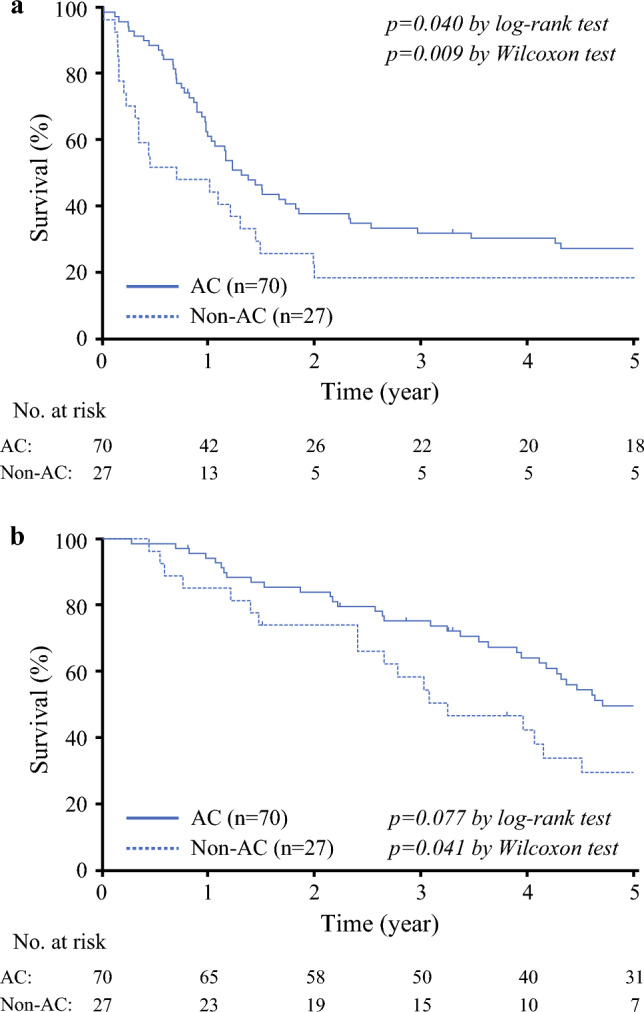
Survival according to adjuvant chemotherapy. (**a**) Recurrence-free survival (RFS). The bold line indicates the RFS curve for the AC group and the dashed line denotes the RFS curve for the non-AC group. (**b**) Overall survival (OS). The bold line indicates the OS curve for the 5-FU group and the dashed line denotes the OS curve for the non-AC group

The RFS curves for patients who received AC and those who did not are depicted in Fig. 2A. In the non-AC group, the 3-year RFS rate was 18.5% in contrast to 32.0% in the AC group (*p* = 0.040 in the log-rank test; *p* = 0.009 in the Wilcoxon test).

The OS curves for patients who received AC and those who did not are illustrated in Fig. 2B. The 3-year OS rates were 58.5% for the non-AC group and 75.3% for the AC group (*p* = 0.077 using the log-rank test; *p* = 0.040 using the Wilcoxon test).

The details of recurrence after PALN resection in the non-AC and AC groups are presented in Supplementary Table 1. Recurrence in distant nodes was the most common pattern, which was followed by lung metastasis in both groups. The distribution of recurrence sites was similar between the two groups. The treatments for recurrence after PALN resection are summarized in Supplementary Table 2. Although many patients received systemic therapy, the frequency (61%) was lower than that in the AC group (86%, *p* = 0.035).

### Subgroup Analyses for Survival Benefit Provided by AC

**Fig. 3:**
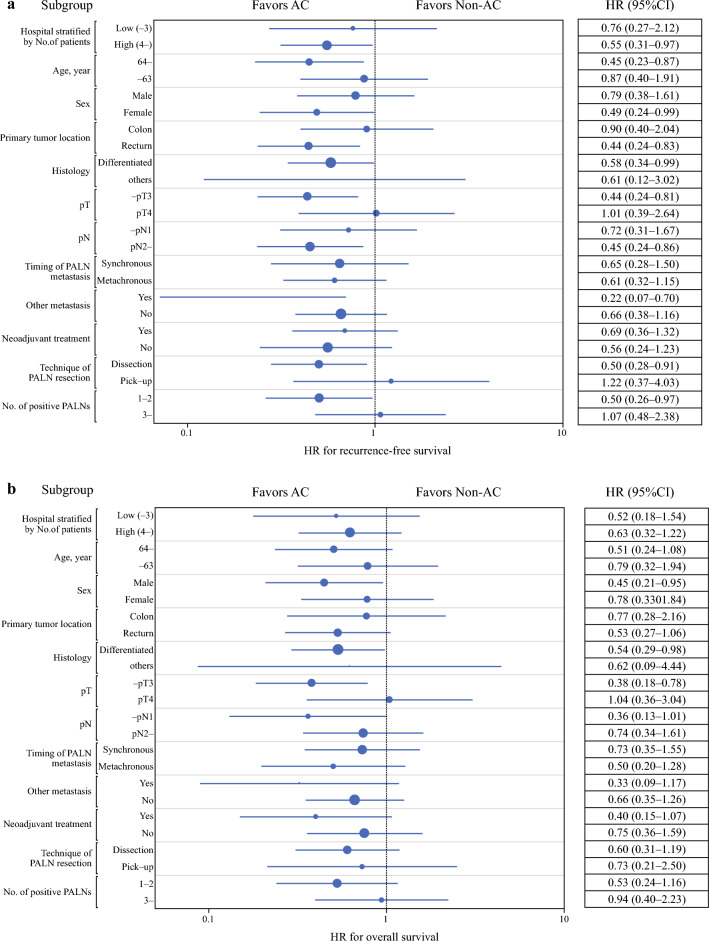
Forest plot of survival based on adjuvant chemotherapy in the selected subgroups. (**a**) Forest plot of recurrence-free survival (RFS). Hazard ratios with 95% confidence intervals are shown. (**b**) Forest plot of overall survival (OS). Hazard ratios with 95% confidence intervals are shown *HR* hazard ratio, *CI* confidence interval, *Differentiated* differentiated adenocarcinoma, *PALN* paraaortic lymph node, *AC* adjuvant chemotherapy

Subgroup analyses were performed to determine the survival benefit of AC. As shown in Fig. 3A, the effect of AC on reducing the risk of recurrence was heterogeneous across subgroups, i.e., AC provided RFS benefit in case of female sex, age ≤ 64 years, rectum, differentiated histology, -pT3, pN2-, presence of other distant metastasis, dissection of PALNs, and 1–2 PALN metastases. In contrast, the advantage of the implementation of AC in OS was observed in male patients, CRC of differentiated histology, -pT3, and -pN1 (Fig. 3B). Notably, hazard ratios for both RFS and OS were approximately 1 in patients with pT4 CRC and in those with ≥ 3 PALN metastases (Fig. 3A and B).

### Comparison of Baseline Characteristics and Prognosis According to AC regimen

Similar to the comparison between the non-AC and AC groups, baseline characteristics of the 5-FU and L-OHP groups were compared. As shown in Table 2, no significant intergroup difference was observed in any of the clinicopathological parameters linked to primary CRC and PALN metastasis.

**Table 2:** Baseline characteristics of patients according to adjuvant chemotherapy regimen after paraaortic lymph node resection

Variable		5-FU (*n* = 14)	L-OHP (*n* = 52)	*p*
Hospital	Low-volume center	3 (21%)	12 (23%)	1.00
	High-volume center	11 (79%)	40 (77%)	
Age, year	Median (IQR)	64 (55–69)	62 (52–67)	0.41
Sex	Male	7 (50%)	32 (62%)	0.64
Age, year	Median (IQR)	64 (55–69)	62 (52–67)	0.41
Sex	Male	7 (50%)	32 (62%)	0.64
	Female	7 (50%)	20 (38%)	
Primary tumor location	Colon	8 (57%)	22 (42%)	0.49
	Rectum	6 (43%)	30 (58%)	
Histology	Differentiated	14 (100%)	46 (88%)	0.33
	Others	0 (0%)	6 (12%)	
pT	pT3	6 (43%)	27 (52%)	0.51
	pT4a	7 (50%)	15 (29%)	
	pT4b	1 (7%)	10 (19%)	
pN	pN0	1 (7%)	2 (4%)	0.45
	pN1	5 (35%)	7 (13%)	
	pN2	4 (29%)	17 (33%)	
	pN3	4 (29%)	26 (50%)	
Timing of PALN metastasis	Synchronous	6 (43%)	38 (73%)	0.070
	Metachronous	8 (57%)	14 (27%)	
Other metastasis at initial presentation	None	9 (64%)	44 (85%)	0.19
	Other distant lymph nodes	0 (0%)	1 (2%)	1.00
	Liver	1 (7%)	5 (10%)	1.00
	Lung	0 (0%)	1 (2%)	1.00
	Peritoneum	1 (7%)	2 (4%)	0.52
	Ovary	0 (0%)	1 (2%)	1.00
Neoadjuvant treatment for PALN metastasis	No	11 (79%)	41 (79%)	1.00
	Yes	3 (21%)	11 (21%)	
Technique of PALN resection	Dissection	6 (43%)	28 (54%)	0.67
	Pick-up	8 (57%)	24 (46%)	
Number of positive PALNs	Median (IQR)	2 (1-5)	1 (1-3)	0.52
Complications after PALN resection *	No	12 (86%)	45 (87%)	1.00
	Yes	2 (14%)	7 (13%)	

^*^ Clavien-Dindo classification grade 3 or higher

*5-FU* 5-fluorouracil, *L-OHP* oxaliplatin, *IQR* interquartile range, *Differentiated* differentiated adenocarcinoma, *PALN* paraaortic lymph node, *N/E* not evaluated

**Fig 4:**
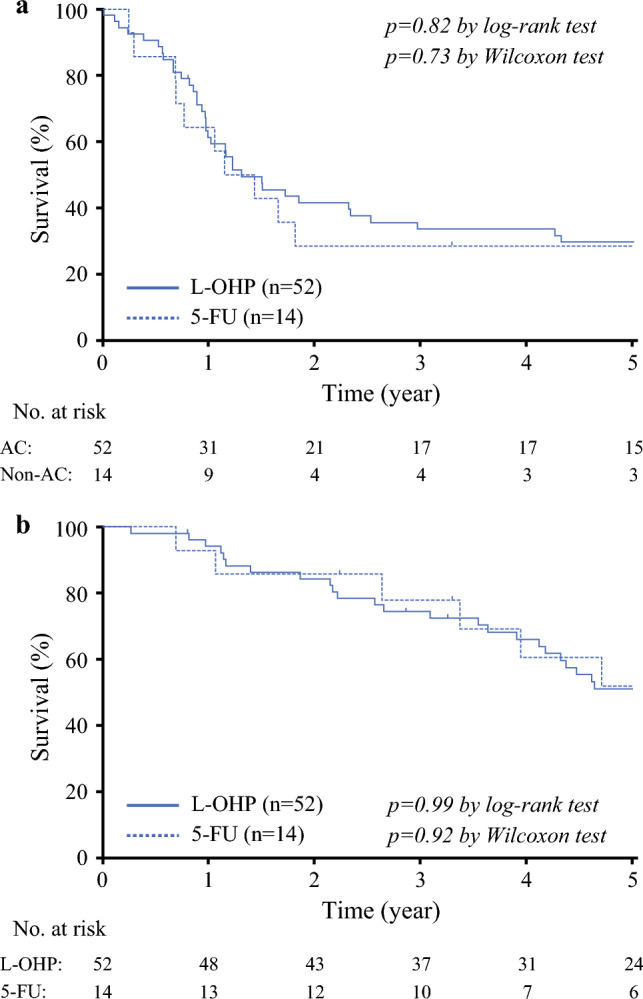
Survival according to adjuvant chemotherapy regimen. (**a**) Recurrence-free survival (RFS). The bold line indicates the RFS curve for the L-OHP group, and the dashed line denotes the RFS curve for the 5-FU group. (**b**) Overall survival (OS). The bold line indicates the OS curve for the L-OHP group, and the dashed line denotes the OS curve

The RFS curve was compared between patients receiving L-OHP-based AC and those receiving 5-FU monotherapy. As presented in Fig. 4A, the L-OHP group exhibited a higher RFS curve after two postoperative years, but the difference was not significant (*p* = 0.82 using the log-rank test; *p* = 0.73 using the Wilcoxon test). The 3-year RFS rates were 28.6% and 33.5% for the 5-FU and L-OHP groups, respectively.

The OS curves for patients who received L-OHP and 5-FU monotherapy are illustrated in Fig. 4B. The curves overlapped throughout the follow-up period (*p* = 0.99 using the log-rank test; *p* = 0.92 using the Wilcoxon test). The 3-year OS rates were 77.9% and 74.5% for the 5-FU and L-OHP groups, respectively.

The details of recurrence after PALN resection in the 5-FU and L-OHP groups are shown in Supplementary Table 3. Patients in the 5-FU group were more likely to develop lung metastasis, whereas recurrence in distant nodes was more common in the L-OHP group. Nevertheless, significant differences in recurrence sites were not noted between the two groups. The treatments for recurrence after PALN resection are summarized in Supplementary Table 4. The treatment selection was similar between the two groups, with systemic therapy being the most common (80%–86%).

## Discussion

Surgical resection for colorectal PALN metastasis is still a relatively uncommon approach especially in Western countries. Meanwhile, the issue of which patients with metastasized PALNs from CRC are suitable for surgical resection despite the possibility of complications has been discussed over the last few decades. Although there remains a lack of evidence for the management of PALN metastasis, recent meta-analyses have indicated that select patients may obtain survival benefit under certain conditions.^20,21^ Earlier studies have reported several prognostic factors after PALN resection, which include a small number of metastasized PALNs,^10–12,15,17^ low CEA level,^10^ differentiated histology,^17^ and the absence of metastasis other than PALNs.^11^ However, the implementation of AC has not been listed as an independent prognostic factor after the removal of PALN metastasis in previous retrospective studies.^14,16–18,22,26^ In this study, almost 100 patients who underwent PALN resection at multiple centers were investigated, which showed that AC was linked to favorable prognosis.^23^ Namely, RFS and OS were better in patients with CRC who had received AC than in those who had not undergone AC treatment after PALN curative resection. Therefore, all preceding single-center studies may be underpowered to detect the prognostic difference owing to a small sample size.

The impact of AC has been adequately researched in colorectal liver metastasis. According to the FFCD ACHBTH AURC 9002 study^27^ and Hasegawa et al.,^28^ AC involving 5-FU plus calcium folinate prolonged RFS in patients who underwent hepatectomy for colorectal liver metastasis; however, the regimen failed to improve OS in both trials.^27,28^ The prolongation of OS by AC after PALN metastasis from CRC in the current study cohort could partly be attributed to the fact that systemic therapy was more common compared with the non-AC group. Alternatively, the finding could be specific for AC after surgery for PALN metastasis.

In the subgroup analyses performed in this study, only patients with primary CRC of differentiated histology or -pT3 received both RFS and OS benefits from AC. Thus, AC may be recommended for patients with these pathological features after surgery for PALN metastasis. In contrast, patients with pT4 CRC and those with ≥ 3 PALN metastases did not benefit from this approach. Notably, dissection as the method for resecting metastasized PALNs may be favorable to obtain relapse prevention benefits of AC, as the RFS hazard ratio in the counterpart subgroup (pick-up) was over 1. The advantages of AC were inconsistent or not significant across other subpopulations and showed wide confidence intervals in the forest plots. Owing to the limited number of patients, quantitative interactions between AC and clinicopathological factors remain to be elucidated.

The proportion of L-OHP-containing AC regimens varied widely (6%–66%) among previous studies on PALN metastasis from CRC, and none of these studies examined the effect of L-OHP on survival.^17,18,26^ In this study, the addition of L-OHP to 5-FU did not alleviate recurrence risk or death after PALN resection in patients with CRC. However, the small number of patients in the 5-FU group could have led to type II errors in the survival analyses. In the JCOG0603 study that compared adjuvant mFOLFOX6 and surgery alone after hepatectomy for liver metastasis in CRC, AC considerably improved RFS. Nonetheless, the mFOLFOX6 group exhibited poorer OS than the surgery alone group, without a significant level (hazard ratio 1.35).^29^ The EPOC trial reported improvement in RFS with perioperative FOLFOX4 in colorectal liver metastasis; however, the treatment did not prolong OS.^30^ No RCT has so far compared L-OHP-containing AC and 5-FU monotherapy directly in patients with liver metastasis or other organ metastasis from CRC. Nonetheless, the lack of obvious improvement in OS with L-OHP after PALN resection observed in this study seems to agree with the abovementioned results. The recommendation of AC for patients with CRC after resection of distant metastases other than liver metastasis differs among the major guidelines. While the JSCCR guidelines weakly recommend AC,^31^ the European Society for Medical Oncology states that AC for stage IV CRC is not a part of standard care owing to insufficient evidence.^32^ The National Comprehensive Cancer Network guidelines indicate 5-FU monotherapy or L-OHP-containing doublet therapy after the resection of metachronous metastasis in distant organs other than the liver and lung.^33,34^ Whether 5-FU monotherapy or L-OHP-containing doublet therapy should be recommended as AC after PALN resection in CRC remains to be determined. Prospective studies using a larger cohort should be conducted in the future to shed light on this aspect.

There still remains a paucity of high-level evidence of superiority of surgical resection over non-surgical management for PALN metastasis from CRC. Our study group initially collected patients with colorectal PALN metastasis from 36 hospitals regardless of treatment strategy before 2015. Ideally, the cohort in this study should be compared with those who had never received surgical treatment to address the significance of PALN resection. However, there must be intergroup biases in the extent of PALN metastasis, other organ metastases, and baseline characteristics, such as age and comorbidity, in such comparison. Moreover, a substantial number (27%, 26 of 97) of patients received preoperative chemotherapy in our cohort, which may also hinder appropriate evaluations of the benefits of surgery for PALNs over systemic treatments alone. As systemic therapies have improved dramatically over the last decade, there is a possibility that non-surgical management may now provide comparable outcomes to PALN resection in CRC.

This study has several limitations. First, this research is a retrospective study that examined patient data collated from many hospitals over a 5-year period. Hence, the results may be fraught with heterogeneity problems in surgical and medical treatment strategies. Moreover, all patients with false-positive PALN involvement were excluded during case selection. Second, as mentioned above, significant differences were observed in the technique of PALN resection and implementation of neoadjuvant and post-recurrence treatments after PALN resection between the non-AC and AC groups. Third, there was no standard follow-up protocol after resection of colorectal PALN metastasis. The details of AC, including the timing of initiation, relative dose intensities of chemotherapeutic drugs, duration, and adverse events, were not available. Detailed information on subsequent treatments after recurrence following PALN resection, including the regimens of and responses to systemic therapies administered, could not be collected. Moreover, the database lacked genetic variables, such as *ras*, *braf*, and microsatellite instability status. Finally, performance status and comorbid physical and psychosocial conditions of the enrolled patients that could have possibly affected the treatment selection were unknown.

To conclude, the findings from this study suggested that AC may be associated with favorable RFS and OS in patients with CRC after resection of metastasized PALNs. L-OHP-containing AC could not further improve the survival in these patients. Given the retrospective nature of this study, it is desirable to conduct prospective randomized trials comparing non-AC and AC as well as 5-FU monotherapy and L-OHP-based AC in CRC with PALN metastasis to confirm the current findings.

## Supplementary Information

Below is the link to the electronic supplementary material.

Supplementary file1 (DOCX 31 kb)
