# Supplementary material for: Role of Adjuvant Chemotherapy After Surgical Resection of Paraaortic Lymph Node Metastasis from Colorectal Cancer—A Multicenter Retrospective Study
Source: Ann Surg Oncol. 2024 Nov 18;32(4):2282–91. doi: 10.1245/s10434-024-16537-6 (PMC11882702; doi:10.1245/s10434-024-16537-6)
Supplement: Supplementary file 1 — Supplementary file1 (DOCX 31 kb) [file 10434_2024_16537_MOESM1_ESM.docx]

| **SUPPLEMENTARY TABLE 1** Recurrent sites after paraaortic lymph node resection according to adjuvant chemotherapy | | | |
| --- | --- | --- | --- |
| Recurrent organ | Non-AC (n=27) | AC (n=70） | p |
| Distant lymph nodes | 11 (41%) | 27 (39%) | 0.84 |
| Liver | 5 (19%) | 10 (14%) | 0.84 |
| Lung | 8 (30%) | 22 (31%) | 0.94 |
| Bone | 0 (0%) | 6 (9%) | 0.18 |
| Peritoneum | 2 (7%) | 9 (13%) | 0.72 |
| Ovary | 0 (0%) | 2 (3%) | 1.00 |
| Local | 1 (4%) | 3 (4%) | 1.00 |
| Skin | 0 (0%) | 1 (1%) | 1.00 |
| None | 4 (15%) | 20 (29%) | 0.25 |

Abbreviation: AC, adjuvant chemotherapy.

| **SUPPLEMENTARY TABLE 2** Treatment for recurrence after paraaortic lymph node resection according to adjuvant chemotherapy | | | |
| --- | --- | --- | --- |
| Treatment | Non-AC (n=23) | AC (n=50） | p |
| Surgery | 4 (17%) | 10 (20%) | 0.95 |
| Systemic therapy | 14 (61%) | 43 (86%) | 0.035 |
| Radiotherapy | 3 (13%) | 3 (6%) | 0.37 |
| Best supportive care alone | 2 (9%) | 1 (2%) | 0.23 |
| Unknown | 2 (9%) | 1 (2%) | N/E |

Abbreviation: N/E, not evaluated.

| **SUPPLEMENTARY TABLE 3** Recurrent sites after paraaortic lymph node resection according to adjuvant chemotherapy regimen | | | |
| --- | --- | --- | --- |
| Recurrent organ | 5-FU (n=14） | L-OHP (n=52） | p |
| Distant lymph nodes | 5 (35%) | 18 (35%) | 0.81 |
| Liver | 2 (14%) | 9 (17%) | 1.00 |
| Lung | 6 (43%) | 15 (29%) | 0.50 |
| Bone | 2 (14%) | 3 (6%) | 0.29 |
| Peritoneum | 1 (7%) | 7 (13%) | 1.00 |
| Ovary | 1 (7%) | 1 (2%) | 0.38 |
| Local | 0 (0%) | 3 (6%) | 1.00 |
| Skin | 0 (0%) | 1 (2%) | 1.00 |
| None | 4 (29%) | 16 (31%) | 0.87 |

Abbreviations: 5-FU, 5-fluorouracil; L-OHP, oxaliplatin.

| **SUPPLEMENTARY TABLE 4** Treatment for recurrence after paraaortic lymph node resection according to adjuvant chemotherapy regimen | | | |
| --- | --- | --- | --- |
| Treatment | 5-FU (n=10） | L-OHP (n=36） | p |
| Surgery | 2 (20%) | 6 (17%) | 1.00 |
| Systemic therapy | 8 (80%) | 31 (86%) | 0.64 |
| Radiotherapy | 1 (10%) | 1 (3%) | 0.39 |
| Best supportive care alone | 0 (0%) | 1 (3%) | 1.00 |

Abbreviations: 5-FU, 5-fluorouracil; L-OHP, oxaliplatin.
